# Increase in Pneumococcus Macrolide Resistance, USA

**DOI:** 10.3201/eid1605.091424

**Published:** 2010-05

**Authors:** Lauri A. Hicks, Dominique L. Monnet, Rebecca M. Roberts

**Affiliations:** Centers for Disease Control and Prevention, Atlanta, Georgia, USA (L.A. Hicks, R.M. Roberts); European Centre for Disease Prevention and Control, Stockholm, Sweden (D.L. Monnet)

**Keywords:** Antimicrobial drug resistance, Streptococcus pneumoniae, pneumococcus, macrolides, bacteria, United States, letter

**To the Editor:** Jenkins and Farrell reported an increase in the proportion of macrolide-resistant *Streptococcus pneumoniae* isolates in the United States (*1*). They mentioned increased use and inappropriate prescription of macrolides as potential explanations for the increase in macrolide resistance and expressed doubts, stating “which (if any) of these factors might explain the trends here are not clear.” Although the spread of antimicrobial drug resistance is a complex issue with many contributing factors, we believe that the role of macrolide use should not be understated.

Several studies in Europe have provided evidence for a relationship between macrolide use and resistance. Macrolide exposure leads to emergence of macrolide resistance on the individual level, and countries in Europe with higher outpatient sales of macrolides have more macrolide-resistant pneumococci (*2*).

Outpatient antimicrobial drug use in the United States has decreased since 1995–1996, especially among children. However, use of azithromycin increased in children, and use of macrolides increased in older patients from 1995–1996 through 2005–2006 (*3*). In this context, it would be surprising that after this increase, pneumococci would show different characteristics in the United States than in Europe. A 2001 study showed that increased macrolide use in the United States during 1995–1999 coincided with a doubling of the proportion of macrolide-resistant pneumococci (*4*), and further increases in macrolide use since 1999 (*3*) have contributed to the increase in macrolide-resistant pneumococci.

Decreased macrolide use has led to a decrease in macrolide-resistant pneumococci. A yearly seasonal reduction in antimicrobial drug prescribing in Israel was associated with a decrease in the proportion of antimicrobial drug–resistant pneumococci that caused acute otitis media (*5*). With the introduction of expanded-valent pneumococcal conjugate vaccines, there is promise that drug-resistant pneumococcal disease can be reduced. Nevertheless, judicious use of antimicrobial drugs and a decrease in unnecessary prescriptions, as promoted by the Get Smart: Know When Antibiotics Work (www.cdc.gov/getsmart) campaign, are essential to limiting selection and spread of antimicrobial drug resistance.
